# What Do Adults Know and Think About the United Kingdom Physical Activity Guidelines, and How Should They be Communicated? A Qualitative Exploration

**DOI:** 10.1002/gin2.70066

**Published:** 2026-03-12

**Authors:** Gwen Gillham, Graham Baker, Paul Kelly, Chloë Williamson

**Affiliations:** ^1^ Physical Activity for Health Research Centre University of Edinburgh Edinburgh UK

**Keywords:** advice, communication, exercise, messaging, public health, recommendations

## Abstract

**Introduction:**

Currently, there is limited insight into public understanding and opinion of physical activity guidelines (PAG) despite the acknowledged importance of communicating such health‐related information. This study aimed to explore adults' understanding and opinions of current UK Chief Medical Officer's PAG (UKCMOPAG) in addition to messaging preferences to inform effective communication of future PAG.

**Methods:**

Qualitative semi‐structured one‐to‐one interviews were conducted with 17 adults (52% female, mean age 33.1 years) in Scotland, United Kingdom. The interview schedule included open‐ended questions developed to explore understanding and opinion of guidelines, and preferences for communication using the physical activity messaging framework. Data were analysed using framework analysis.

**Results:**

Participants were aware that PAG likely existed but could not accurately recall them. Once informed of the UKCMOPAG, they found them unappealing and unclear. They expressed uncertainty about the intended audience and questioned their relevance across different groups. Participants were, however, aware of some benefits of PA and had lived experience of short‐term benefits (e.g., improved sleep and mood). As such, there was a preference for communicating UKCMOPAG through positive, relatable messages emphasising everyday benefits. Participants disliked threshold messages, suggesting continuum‐based messages with a sliding scale of PA volume/intensity and associated benefits. Lastly, participants indicated that messages should be delivered by trustworthy sources, identified as the National Health Service (NHS).

**Conclusion:**

This study is the first qualitative exploration of how the general adult population in Scotland interpret the UKCMOPAG. Findings show that current communication strategies are not aligned to public understanding and opinions of PAG, indicating a need to change communication approaches. Future updates should separate technical guidance from public‐facing messages, use relatable language, inclusive examples and ensure delivery by trusted sources. Continuum‐ and gain‐framed messaging highlighting short‐term PA benefits may also improve engagement. Further research is needed to evaluate the effectiveness of such audience‐centred messages.

## Summary

- This is the first qualitative study to explore how adults in the general population in Scotland understand and interpret the content of the UK Chief Medical Officers' Physical Activity Guidelines (UKCMOPAG), identifying a clear misalignment between current communication approaches and public needs.
- When explored qualitatively, awareness and accurate recall of the UKCMOPAG is very low among adults in Scotland, with most participants unable to recall recommended amounts, even when they were aware that guidelines existed.
- Once informed of the UKCMOPAG, adults found them unclear, overly clinical and unsuitable for public audiences, with terms like ‘moderate’ and ‘vigorous’ causing confusion.
- Adults indicated a preference for physical activity messages focused on short‐term, relatable benefits (e.g., improved mood and sleep) grounded in lived experience, rather than long‐term disease prevention or threshold‐based targets.
- Continuum‐based messaging (communicating incremental benefits across a sliding scale of physical activity volume and intensity) was suggested as an alternative over fixed threshold messages, which were perceived as demotivating, particularly for less active individuals.
- Participants expressed a preference for positive, gain‐framed messages using inclusive language, relatable examples and clear guidance on what ‘counts’ as physical activity, including for strength training.
- The National Health Service (NHS) was identified as a key trusted source for physical activity messaging; government‐branded materials were met with scepticism.
- Public‐facing physical activity messages should be developed as a distinct, separate process from technical guideline development, co‐designed with end‐users and targeted to specific population subgroups.
- Future updates to UKCMOPAG communication should apply evidence‐based frameworks such as the Physical Activity Messaging Framework to ensure audience‐centred, effective public communication.

## 1 Introduction

Globally, one in four adults fail to meet World Health Organization physical activity (PA) recommendations [1, 2], contributing to 8% of all‐cause mortality [3]. Addressing inactivity requires a systems approach [4] within which public education and communication are key components [5]. Physical activity guidelines (PAG) are evidence‐based recommendations on the types, amounts and intensities of PA for health benefits. Designed for use by policymakers, practitioners and researchers, PAGs are used to inform public education resources [6] to enhance understanding across multiple levels [4].

In the United Kingdom (UK), the Chief Medical Officers (CMOs) released updated national PAG in 2019 [7], which were intended to be disseminated to the public primarily through health professionals. However, many people lack regular contact with health professionals, and PA is not always discussed when there is contact [8]. Furthermore, few public‐facing messages based on the UK Chief Medical Officers Physical Activity Guidelines (UKCMOPAG) exist, potentially contributing to low public knowledge of the guidelines, with awareness rates reported between 4% and 18% [9, 10, 11]. Similar challenges regarding low awareness and knowledge of PAG are evident globally [12, 13, 14]. Alongside the fact that PA levels are not improving globally [2], these figures indicate that PA communication approaches used to date have been largely ineffective.

Understanding what populations know and think about the guidelines is important for informing communication approaches. Inconsistent definitions and applications of terms such as ‘awareness’ and ‘knowledge’, and a lack of harmony across quantitative measurement methods [9, 10, 11], make it challenging to determine what the public truly know and think of such guidelines [13]. Exploring this in more depth is crucial given the influence of perceptions and motivations on engagement [15]. There is a paucity of qualitative research in this area, despite its potential to improve communication approaches [16, 17].

The ‘UK CMOs’ physical activity guidelines communications framework' advocates for formative research to explore the perspectives and attitudes of the public towards such guidelines [6]. Such insights are required to inform the communication of future versions of PAG through enhanced dissemination of public‐facing messages translated from PAG, as has recently been conducted in Ireland in 2024 [18]. Therefore, this study aims to gather rich qualitative data to explore public understanding and opinions (i.e., what they know and think) of the current PAG in the United Kingdom, and preferences for future guideline communication. To address this aim, the following research questions (RQs) were developed: (1) What is the understanding and opinion of adults in Scotland of the current UKCMOPAG? And (2) What do adults in Scotland consider important for future effective communication of PAG?

## 2 Methods

### 2.1 Study Design

This qualitative study used semi‐structured interviews and adhered to the Standards for Reporting Qualitative Research (SRQR) [19]. Ethical approval was obtained from the University of Edinburgh on 15th June 2023 (study ID S000646).

### 2.2 Participants and Recruitment

This study focused on adults aged 19–64 years (the broadest UKCMOPAG group). Participants were recruited from Scotland and limited to English‐speaking adults only, as evidence supports the creation of targeted and context‐specific messages that are relevant to a particular population subgroup [16, 20, 21]. Convenience sampling involved promoting the study on multiple social media platforms, and snowball sampling was employed to attempt to address representation by encouraging participants to share study details with less active individuals, aiming to include diverse PA levels [22].

### 2.3 Sample Size

The concept of information power [23] informed the sample size. This approach considers the relevance of factors such as study aim, sample specificity and analysis strategy within the study context, ensuring the sample size is sufficient to provide meaningful insights while enhancing study rigour [24, 25, 26]. Specifically, as our study had a narrow aim, applied the existing theory‐informed Physical Activity Messaging Framework (PAMF), featured strong quality of dialogue in data collections and involved participants that held specific shared characteristics, a sample size of 15–20 was deemed appropriate to address the aim.

### 2.4 Data Collection

Participants reviewed study information and provided consent online (Qualtrics™, Provo, UT). One‐to‐one interviews were conducted and recorded via Microsoft Teams from June to July 2023 by G.G. Demographic data (age and gender) were collected at the outset. Interviews were transcribed verbatim, pseudonyms applied and data securely stored on a University of Edinburgh OneDrive.

The interview schedule, developed using the framework for semi‐structured interview guides in qualitative research, developed by Kallio et al [27], consisted of three sections that explored understanding and opinion of UKCMOPAG and suggested changes to national guideline communication. The full interview schedule is shown in Supporting Information: Appendix SA.

Key terminology used in this schedule, and throughout this study, is shown in Table 1. Participants were informed of current UKCMOPAG infographic content only after assessing their understanding. The schedule was pilot tested, and an iterative process allowed minor amendments to enhance data collection.

**Table 1:** Definitions of key terms.

Term	Definition
Understanding	Understanding of UKCMOPAG was recognised as inclusive of both awareness and knowledge of guideline content. This was to account for the possibility that guideline content had been communicated, such as via healthcare professionals, in a way that meant the participant was unaware of UKCMOPAG existence, but knowledgeable of information contained within the UKCMOPAG.
UKCMOPAG	Considered to be the information contained within the UKCMO PAG infographic (Supporting Information: Appendix SB). Content includes 150 min moderate activity OR 75 min of vigorous activity, 2 days of strength activity, ‘every minute counts’ and ‘some is good, more is better’ as key recommendations. The content of the UKCMO PAG infographic has been assumed as the information currently prioritised for communication.
Opinion	Opinion of UKCMOPAG accounts for participants views of the current UKCMOPAG infographic content.
Future communication	Suggestions for change extends beyond the current infographic, considering possible adaptations or innovations that most resonate with participants.

### 2.5 Data Analysis

Interview transcripts were analysed by G.G. to generate initial codes and develop themes. A combination of deductive and inductive thematic development [28] enabled integration of insights from both the UKCMOPAG and relevant literature [29].

Three hierarchical themes were preidentified in line with the two RQs: (1) understanding of UKCMOPAG, (2) opinions of these and (3) suggested changes to communication. Subthemes related to RQ2 were preidentified, corresponding to the three sections of the PAMF [15]: message aim, message content and message format and delivery.

Framework analysis [30, 31] was employed, allowing integration of structured analytical procedures with subjective interpretation. This involved the systematic application of a matrix to preserve connections to individual participant views, following a seven‐step process [32].

To enhance rigour, a ‘critical friends’ approach was applied whereby two independent researchers, C.W. and a postgraduate student, each coded transcript samples (*n* = 4) and discussed codes with G.G. This process improved reflexivity and helped mitigate potential bias [33, 34]. Furthermore, G.G. maintained a reflexive journal throughout data collection and analysis, documenting decision‐making processes. The initial coding framework was developed by G.G. and iteratively reviewed by C.W. This collaborative approach, combined with adherence to Framework Analysis [32] protocols and SRQR standards [19], ensured methodological rigour despite single‐researcher data collection, which is appropriate and common for qualitative studies of this scope, and helps maintain consistency [35].

## 3 Results

Twenty‐one individuals engaged with the Qualtrics recruitment survey, with 17 (81%) providing consent and completing interviews (mean age 33.1 years, 53% female). In the following section, subthemes are presented beneath the pre‐determined hierarchical themes. The following section is organised by research question. Results are presented with dominant themes in the narrative summaries, and with all subthemes and select illustrative quotes in Tables 2 and 3.

**Table 2:** Participants' understanding and opinion of PAG: Illustrative quotations.

Sub theme	Description of subtheme	Select illustrative quotes
Awareness and recall of guidelines	Highlights that participants were not aware PAG exist, that they have not been provided this information by HCP and have correct and incorrect recall of aspects of the PAG.	‘I don't think I have ever had any exercise advice specifically or seen any public sort of stuff, not even from a health care professional’ (P1, Harold, 27) ‘Oh 10,000 steps maybe, I can't think of anything else specific’ (P4, Ailsa, 54 F) ‘I don't hear very much nowadays it's all just social influencers posting their stuff I guess’ (P5, George, 32 M) ‘There's guidance for everything I'm sure there is, but I just don't know what they are’. (P9, Sandra, 27 F) ‘I think I'm aware that there should be guidelines on these types of things but it's not ever something I've looked into’ (P11, Joanna, 26 F)
Awareness of benefits of being active and risks of being inactive	Highlight that participants mainly understood PA benefits through personal experience, focusing on mental well‐being and day‐to‐day improvements. Long‐term health outcomes/risks felt less personally relevant for some.	‘I really would not want to have dementia, so yeah I know that running every day is investing in my future as much as I'm getting benefits today from it as well‘ (P2, Katie, 32) ‘With activity there is a kind of space where you get into where you're processing your day a bit, I think there's a sort of mindfulness with that’ (P3, Kyle, 27 M) ‘The disadvantages… you're not going to be a healthy… but I don't really know, I just know you're not going to be healthy’. (P6, Kimberly, 27 F) ‘All of it comes back to mental health I think’ (P8, Luke) ‘I know that when I exercise, I sleep better and it improves my mental health, but that's come from lived experience versus like public health information’ (P11, Joanna, 26 F) ‘It reduces the chance of certain illnesses, obesity, cancer, heart disease and then mental health benefits as well’ (P17, Connor, 28M)
Perception of frequency, duration and intensity	Highlights that participants felt that PA should be regular, and believed higher intensity was more beneficial. Most overestimated the recommended weekly duration and were unclear on terms ‘moderate’ and ‘vigorous’.	‘[physical activity is] anything that gets my heart rate up for a reasonable amount of time… and I think upwards of at least 20 minutes’ (P1, Harold, 27) ‘My understanding would be that the higher intensity would benefit your health more otherwise why do we do it’ (P5, George, 32 M) ‘Everyday… 20 minutes or 2 hours… it's just about moving your body really’. (P13, Lisa, 26 F) ‘If it's short, you'd probably need more intensity’ (P14, Isaac, 26 M) ‘I can't even imagine you only getting 150 a week, I can't imagine you getting anything from that I mean… that's like 20 minutes a day’ (P15, Kathryn, 58 F)
Trust and credibility issues	Highlighted that participants were often sceptical of official PAG, questioning their credibility. Some saw them as politically or commercially driven, leading mistrust.	‘I think that is there a difference between what official guidelines state and what you actually should be doing’ (P3, Kyle, 27 M) ‘I don't think a lot of people would look at that and go ‘I must improve’… people would take it with a pinch of salt’ (P8, Luke, 29 M) ‘The Scottish government… it's in their interest so you know there's 100% bias there and the information might be entirely false’ (P11, Joanna, 26 F) ‘It's probably more commercially motivated‘ (P12, Grant, 38 M)
UKCMOPAG are unclear and unsuitable for the public	Highlighted that participants found UKCMOPAG to be confusing, with difficulty interpreting what counts. Many felt they were too general, not reflecting individual needs or lifestyles.	‘How do I know if I've done the right intensity, how do I know if I've done the right type of exercise?’ (P3, Kyle, 27 M) ‘Do you need to be doing your 150 minutes separate from your 60 minutes… do I need to do 60 minutes then plus 30 minutes then plus strength training or what?‘ (P9, Sandra, 27 F) ‘150 minutes – that sounds like a lot, but when you think about it split over 7 days its better’. (P13, Lisa, 26 F) ‘It's almost like an out‐of‐control kind of thing… quite harsh term like ‘vigorous’… almost as in… you could be hurting yourself’ (P16, Caroline, 25 F) ‘If someone said to me to be doing three sessions of vigorous exercise, I'd be like what does that mean? I don't know’. (P16, Caroline, 25 F)

**Table 3:** Participants' suggestions for future communication of PAG: Illustrative quotations.

Subtheme	Subtheme description	Select illustrative quotes
Message aims and context	Highlighted that participants were unclear about the purpose and aim of current PAG (including who the target audience was), and felt that different messages with different aims were required for different population groups.	‘19‐year‐old is very different from [a] 64‐year‐old’ (P14, Isaac, 26 M). ‘I guess if what you're trying to do is move people from zero, I can see that there might need to be different messaging for different groups of people’ (P7, Alistair 55 M) ‘I suppose it's like who's the message for… probably the ones that are sitting on the sofa… or the people with mental health issues who are really struggling’ (P10, Emily, 26 F) ‘I suppose it's about having the resources in place to allow people to make those choices’ (P13, Lisa, 26 F)
Message content	Highlighted that participants preferred messages that focused on short‐term, relatable benefits and avoided fear‐based tones. They valued inclusive, tailored content and clearer guidance on terms and resistance training. They supported a flexible, gradual approach over fixed targets.	‘Case studies of people that are really relatable would be more impactful’ (P2, Katie, 32 F) ‘I think it's a good message… to explain the difference between moderate and vigorous but also give people simple ways of doing that’. (P4, Ailsa, 54 F) ‘If they do 15 minutes three times a week and they weren't doing that before then that's a good start… actually once they've done that, they think ‘I can do more of it’ (P12, Grant, 38 M) ‘Going from zero exercise… aim to do 50 to 100 minutes… then your target is 150… would work better than just here's your one number’. (P5, George, 32 M) ‘They don't emphasise enough that young women in particular should be doing resistance‐based training… that's so important for bone density’ (P9, Sandra, 27 F) ‘If you think it's like a full time Mum who also works a job her sitting there going “where am I going to get 150 minutes a week”… if you're saying 20 minutes, most people could probably manage 20 minutes… like a long periods of time people are going to be put off by that I think’ P10, Emily, 26 F ‘I don't think scaremongering is the sort of… “you're more likely to die of a heart attack if you don't exercise”… if you could say “you were going to live a better quality of life,” that's something that I'd want to listen to’ (P11, Joanna, 26 F) ‘I think it's fine to articulate the risks because that will be what prompts some people as long as it's balanced with the benefits as well’. (P12, Grant, 38 M). ‘But minutes… don't find it relatable to me and I'm you know relatively kind of well‐educated articulate and… I kind of get it, but it's not a particularly engaging metric I don't think’ (P12, Grant, 38 M)
Format and delivery	Highlighted that participants saw trusted sources as key to credible PA messaging, favouring the NHS and health professionals over government. Social media added to confusion, unless content was evidence‐based and from reliable sources.	‘It's all a bit rich coming from, you know, your 18 stone doctor that hasn't done an ounce of exercise in 20 years’ (P1, Harold, 27) ‘I mean I just don't know when… some people have never been to the GP’ (P4, Ailsa, 54 F) ‘Social media now is 100% the platform I would present any information… I don't look at anything else’ (P5, George, 32 M) ‘People are suspicious of government and their intentions… it might be preferable for the information to come under the health banner… to come from the NHS’ (P7, Alistair, 55 M) ‘I think in this day and age… there's so many unreliable sources… I don't know whether a few doctors or something need to get a wee TikTok account to get the message out there’ (P9, Sandra, 27 F) ‘We're disadvantaged that there's so much information, like we are overstimulated by information…. soon as I think the source is dodgy… I'm not interested’ (P11, Joanna, 26 F)

Research question 1 What is the understanding and opinions of adults in Scotland of the current UKCMOPAG?

Five subthemes were identified within the pre‐established hierarchal themes of understanding of UKCMOPAG and opinions of UKCMOPAG. Illustrative quotations corresponding to each subtheme are presented in Table 2.

### 3.1 Subtheme 1: Awareness and Recall of Guidelines

Participants' awareness of and ability to recall the UKCMOPAG was generally low. While some correctly recalled specific elements of the UKCMOPAG (such as every movement counts), or incorrectly recalled targets not included in the current PAG (such as 10 000 steps daily), most could not recall current recommendations. Several noted they had never received guidance relating to the PAG from healthcare professionals or seen public health messaging relevant to these. There was widespread uncertainty about where to access government‐administered information, with participants commonly referencing social media as their primary exposure to (non‐guideline‐related) PA messaging. Others expressed a belief that such guidelines must exist, but had never encountered or sought them out.

### 3.2 Subtheme 2: Awareness of Benefits of Being Active and Risks of Being Inactive

Participants showed awareness of benefits of PA, however, this awareness typically came from their own lived experiences. Participants identified the main health benefit as improvement in mental health. Many understood PA to help manage stress, anxiety and low mood and viewed improved mental well‐being as one of the most immediate and meaningful outcomes. Some physical benefits were also recognised, but the risks of inactivity were often seen as abstract or distant. Though some participants were aware of long‐term health‐related outcomes that are highlighted in the UKCMOPAG, such as reduced risk of dementia, diabetes and cancer, these were harder for most participants to connect with on a personal level. Largely, when considering benefits, participants focused on day‐to‐day functional benefits learnt from individual experience (e.g., improved sleep) rather than longer‐term physical benefits.

### 3.3 Subtheme 3: Perception of Frequency, Duration and Intensity

Participants generally felt that PA should occur regularly, with many suggesting it should be daily. However, views varied on what constituted sufficient duration, ranging from any daily movement to a minimum of 20–30 min for health benefits. Participants generally believed that PA had to be of a certain intensity, and that higher‐intensity activity was more beneficial for health, with one participant believing that these could offset longer durations. Though intensity was often measured using indicators such as increased heart rate or ability to maintain conversation, participants were uncertain as to how this related to terms ‘moderate’ and ‘vigorous’. Most thought weekly duration would be much greater than 150 min of moderate activity, and some thought that this was too low to be meaningful.

### 3.4 Subtheme 4: Trust and Credibility Issues

Participants expressed varying degrees of scepticism toward official PAG, often questioning their relevance, credibility and potential impact. Others doubted the motives behind public health messaging, suggesting that it could be biased, politically influenced or commercially driven, particularly if linked to government. There was also a perception that messages may not always be evidence‐based, leading to confusion and disengagement.

### 3.5 Subtheme 5: UKCMOPAG Are Unclear and Unsuitable for Public

Once informed of the PAG, participants commonly described them as confusing or ambiguous. Several struggled to interpret what counts toward the recommended 150 min; whether it should be accumulated in specific session lengths, if it included everyday movement, how it aligned with other advice such as strength training and how to combine ‘moderate’ and ‘vigorous’ durations. Terms such as these were often seen as overly clinical, intimidating or unclear. Some participants expressed difficulty distinguishing between ‘exercise’ and ‘physical activity’, were uncertain about what qualifies and how to track their efforts.

Furthermore, several participants felt that the guidelines were overly generalised and failed to account for individual differences such as age, ability or lifestyle, reducing their perceived usefulness.

Research question 2 What do adults in Scotland consider important for future effective communication of UKCMOPAG?

Three subthemes were identified within the pre‐established hierarchal theme of *suggested changes to the communication of PAG*. Illustrative quotations corresponding to each subtheme are presented in Table 3.

### 3.6 Subtheme 1: Message Aims and Context

Participants expressed mixed views on the current purpose of PAG, with some perceiving them as valuable and informative, while others questioned their usefulness or relevance. Clarity regarding the intended aim of the current PAG—whether to initiate behaviour change among inactive individuals or to support those already active—was viewed as critical to message relevance, and should be clearer in future communication. Participants expressed the belief that in future, different messages with different aims were required for different populations, and that messages should empower people to make their own choice regarding PA behaviour.

### 3.7 Subtheme 2: Message Content

Several participants noted that messages should highlight short‐term, tangible outcomes, such as improved mood, rather than focusing on long‐term disease prevention. Largely, they preferred messages that highlighted the benefits of PA, avoiding fear‐based or blame‐oriented tones.

Participants also emphasised the importance of relatable and inclusive content, such as case studies and examples tailored to diverse groups. For example, some highlighted the need for clearer guidance on resistance training for women, while others suggested that age‐specific messaging would enhance relevance and impact. Clarity around terms like ‘moderate’ and ‘vigorous’ was also deemed necessary.

Participants emphasised the need for PA messages to be realistic and motivating. Some participants felt that a graduated or sliding scale approach, recognising and encouraging incremental progress rather than relying on a single, fixed target, would be preferable. Many found rigid thresholds demotivating or unrealistic, particularly for those starting from low activity levels. Instead, participants generally felt that messaging should promote small, achievable goals that build over time. Daily recommendations were identified as more accessible, though some questioned the relevance of abstract metrics like minutes.

### 3.8 Subtheme 3: Format and Delivery

Participants felt that the credibility of PA messaging was closely tied to the perceived trustworthiness of its source. The NHS was generally viewed as more trusted than the government, which was often associated with political motives. For this reason, participants expressed a preference for guidance by credible health professionals, although noted that such messaging can feel patronising, especially if the messenger does not appear to embody the advice they give. Participants also noted that they rarely visit health professionals.

Concerns were raised about information overload and the challenge of navigating inconsistent or unclear messages, particularly across social media. While social media was recognised as a powerful tool for dissemination, participants stressed that content must be evidence‐based and delivered by trusted figures.

## 4 Discussion

### 4.1 Principal Findings

This is the first qualitative study to explore how adults in the UK general population understand and interpret the content of the current UKCMOPAG, and their preferences for communication of these guidelines. While previous research has reviewed international perceptions of PA and sedentary behaviour guidelines [36] and explored communication preferences among underserved communities in the United Kingdom [37], no study to date has examined how UK adults engage with the content of their own national guidelines. This study therefore provides novel insight into understanding and opinion of the UKCMOPAG.

Our study provides evidence that the approaches used to communicate the most recent PAG in the United Kingdom are not aligned with opinions and preferences of the adults we interviewed. The key findings demonstrate how national guidelines are interpreted by adults, revealing issues with clarity, relevance and accessibility. Our study found that although most participants in our study were aware that guidelines likely existed, they were unable to accurately recall them. Once informed of UKCMOPAG, many described the information as unclear and difficult to relate to. Several participants questioned the purpose of PAG, uncertain about intended use and target audience.

This study also identified clear messaging preferences for how PAG should be communicated in the future. Participants expressed a preference for positive messages with short‐term benefits, such as improved sleep and mood, as this related to their lived experience. Messages with everyday relevance were viewed as more motivating than long‐term health benefits. Incorporating these findings into future national‐level communication campaigns may improve public engagement through audience‐centred approaches, aligning with end‐user understanding and preferences.

### 4.2 Comparison With Wider Literature and Plausible Explanations

This study found that adults in Scotland have limited awareness and recall of the content of UKCMOPAG. These findings align with prior quantitative research, which has predominantly assessed knowledge of recommended duration, with recall rates ranging from 8.4% to 18% in UK samples [9, 10, 11]. The 2013 Scottish Health Survey remains the only prior research to assess both duration and intensity knowledge in a UK sample, finding that only 4% could recall both components [38]. Most participants in our study overestimated the recommended duration, potentially indicating a reliance on personal assumptions and societal influences when seeking advice about being active. Related findings by Knox et al. [39] show that individuals often overestimate their own activity levels in relation to the guidelines. A recent evaluation of the Canadian 24‐h Movement Guidelines for Adults found that despite having a large reach (approximately 11.9 million), this did not translate to knowledge (1.6%) [40]. Though those guidelines differ from the UKCMOPG, this combination of findings highlights further misconceptions and reinforces the need for clearer, relatable communication.

Our findings extend previous research by also providing new, qualitative insights into reasons for this low awareness. Participants reported that PAG were not routinely communicated by health professionals, or that they had limited engagement with healthcare services. This suggests that low awareness may result from both limited communication and infrequent access to trusted sources of health information. Future research could explore alternative delivery of messages beyond traditional healthcare settings.

Further, when informed of PAG, threshold messages were deemed to be demotivating, potentially reducing engagement. This adds to the literature that suggests that threshold framing may have limited effectiveness in promoting PA [41]. A novel finding from this study was the suggestion from participants for continuum‐based messages, which communicate benefits across incremental durations and/or intensities, rather than providing one fixed target. This could include emphasis on the benefits of shorter bouts of PA. Future research should explore the impact of continuum‐based versus threshold‐based messaging.

Public opinion of the UKCMOPAG remains under‐researched. A recent systematic review, which included 31 international studies across age groups, guideline types and stakeholder categories, identified only six that explored perceptions of adult PAG in end‐users [36]. Of these, only one study [37] focused on perception of UKCMOPAG, specifically within underserved populations. Our findings add to this by exploring understanding in a general adult population, highlighting that participants found current guidelines unsuitable as public‐facing communications. This reinforces that public messages should be developed separately from technical guidelines, using language and framing appropriate to the target audience. We also found that adult participants in Scotland wanted clear examples of PA that ‘counts’ towards guidelines, and preferred these not be communicated in minutes—a finding not previously reported. Developing lay‐language alternatives of PAG in hours and minutes with clear examples and accompanying motivational messages is an approach that has been recently adopted by colleagues in Ireland [18]. Though evaluation of this approach has not been conducted, the messages developed are in line with current evidence.

Participants also expressed a preference for positive or gain‐framed messages that highlight the benefits of PA. While this is widely supported by existing literature [41, 42], our findings illustrate that the lived experiences of participants directly inform messaging preferences. Specifically, participants described short‐term affective outcomes, based on prior experience was more meaningful and motivating than long‐term benefits (relating to future health risks of inactivity), which they felt abstract and distant to them. However, there is a scarcity of research evaluating the effects of messages promoting short‐term benefits, and future studies should aim to address this gap.

In terms of message delivery, a key finding from this study was that the messenger or source must be trustworthy and reliable. Specifically, participants noted scepticism and a lack of trust in Government‐branded materials in comparison to the National Health Service (NHS) who they deemed trustworthy. This is in line with broader public health research, particularly following the COVID‐19 pandemic; a systematic review of 41 studies found inconsistency in public trust towards government, but generally consistent trust in health care providers [43]. Future PAG communication strategies should therefore carefully consider who the message is branded or delivered by.

### 4.3 Strengths and Limitations

This novel study employed rigorous qualitative methods, but limitations exist. Demographic data were limited to age and gender, despite evidence that communication preferences may vary by ethnicity and socioeconomic background [11, 14]. Convenience sampling likely generated an active group, underrepresenting the insufficiently active population most in need of intervention. While not statistically representative, our sample provided sufficient information power for exploratory qualitative research, with findings offering transferable insights for similar populations. Future research should explore guideline perceptions and messaging preferences across diverse subgroups, such as segmented by, for example, PA levels, intention to be active, ethnicity and socioeconomic status. While qualitative sample size remains debated [44], use of information power, critical friends and reporting principles (SPQR) were incorporated to enhance the rigour and robustness of the findings.

### 4.4 Implications

Further formative research is required to explore guideline perceptions and messaging preferences in various population segments. This study's participants came from a broad group (adults), and further segmentation is required, with particular focus on inequalities and those groups who could benefit most from improved PA communication. Research is also required to test and evaluate various messages and provide experimental evidence for message effectiveness. Specifically, studies are needed to test and evaluate the impact of delivering threshold versus continuum messages and the effects of messages promoting short‐term benefits of PA in various populations. Future research may also focus specifically on strength and balance guidelines and messages, which featured minimally in the current study, and are a key gap in the research, with participation in strength‐related activity remaining approximately half that of aerobic activity [45].

The UKCMOPAG were published in 2019 [7] with a goal to update in 2029. Evidence, including findings from this study, clearly demonstrates that the way in which future national‐level guidelines in the United Kingdom needs to be carefully considered. Development of public‐facing messages should be considered an essential and separate process from guideline development, and, in line with evidence [15], messages should have a clear target audience and aim. For the adult (public) group specifically, based on our findings, developed messages should focus less on threshold amounts of PA, and more on promotion of short‐term benefits of PA, as well as emphasising the benefit of shorter bouts and lower intensities of PA. Message content should be relatable, language should be lay‐friendly and clear, and messages should be delivered through messengers deemed trustworthy, such as the NHS. More broadly, PA messages targeted to the public end‐users should be developed by applying evidence‐based tools such as the PAMF for public‐facing messages [15] and the UK CMOs Physical Activity Guidelines Communications Framework [6] for messages to professional audiences, who may be key conduits to the public.

Overall, this study's findings have direct implications for the communication, dissemination and implementation of PAG. Our results suggest that top‐down approaches whereby technical guidelines are minimally adapted for public consumption are misaligned with public needs and preferences. Rather, our results add to those of other studies, which as a whole support a paradigm shift: separating technical guidance from public‐facing messages, co‐designing messages with end‐users and employing gain‐framed approaches delivered through trusted channels. While context‐specific to Scotland, these principles are transferable to other nations updating their PAG communication strategies.

## 5 Conclusion

Understanding public knowledge and opinions of PAG is vital for future communication strategies. This study found that adults in Scotland find the UKCMOPAG unappealing and unclear, and have a preference for messages that communicate short‐term benefits of PA rather than threshold amounts of recommended PA. The findings also show messages should be delivered by messengers perceived as trustworthy by adults. Findings of this study should directly inform communication plans of future updates of the UKCMOPAG to ensure more effective PA messaging.

## Author Contribution

**Gwen Gillham:** conceptualisation (equal), formal analysis (leading), investigation (leading), methodology (equal), resources (leading), visualisation (leading), writing – original draft preparation (leading). **Graham Baker:** methodology (supporting), writing – review and editing (supporting), supervision (supporting). **Paul Kelly:** methodology (supporting), writing – review and editing (supporting), supervision (supporting). **Chloë Williamson:** conceptualisation (equal), formal analysis (supporting), investigation (supporting), methodology (equal), writing – original draft preparation (supporting), writing – review and editing (leading), supervision (leading).

## Funding

The authors received no specific funding for this work.

## Conflicts of Interest

The authors declare no conflicts of interest.

## Supporting information

Appendices.

## Data Availability

Full research data are not shared due to ethical restrictions. The full coding framework is available upon reasonable request from the corresponding author.
