# Supplementary material for: What Do Adults Know and Think About the United Kingdom Physical Activity Guidelines, and How Should They be Communicated? A Qualitative Exploration
Source: Clin Public Health Guidel. 2026 Mar 12;3(2):e70066. doi: 10.1002/gin2.70066 (PMC13563404; doi:10.1002/gin2.70066)
Supplement: Supplementary file 1 [file GIN2-3-e70066-s001.docx]

**Appendices**

Appendix A – Interview schedule

| Section | Question | Additional probing questions | Key points of inquiry |
| --- | --- | --- | --- |
| Understanding of UKCMO PAG | Can you tell me about any guidance or guidelines on physical activity that you are aware of? | Can you tell me about any physical activity information that you are aware of?  Where did you hear this information?  Did you know that there are government guidelines? | Encourage discussion of awareness of UKCMO PAG and of any recommendations relating to physical activity. Prompt consideration of source of this information |
|  | What is physical activity? | What does it mean to be active?  What counts as physical activity?  What’s the difference between physical activity and exercise? | Explore understanding of terms used in the UKCMO PAG infographic  Explore understanding of advised duration, frequency and intensity |
|  | Can you describe different intensities of activity? | Can you categorise activity by level of effort?  Does it matter what intensity of activity you do? If yes, why?  What do you consider moderate activity to be? Can you give examples?  What do you consider vigorous activity to be? Can you give examples? |  |
|  | In a week how much physical activity should you do? | How long should you be active for in total each week?  How often should you be active? |  |
|  | Does it matter how long each activity lasts? | Is there a difference between one long activity versus multiple short activities?  What’s the shortest duration of activity that would have a benefit on your overall health?  In what way does duration of activity affect health? |  |
|  | What benefits might you gain from physical activity? | Why is it good to be active?  Can you give examples of health benefits (mental and physical) (short and long term)? | Explore understanding of benefits to being physically active and risks of being inactive, as shown on the UKCMO PAG |
|  | What are the disadvantages to being less physically active than recommended? | How do you think it affects health to be below this recommended amount?  How would it affect you to achieve some activity, but below the recommended amount? |  |
| Opinion of UKCMO PAG | Do you think that knowing the guidelines encourages you to be more active? | Do you find physical activity guidelines motivating?  Would knowing the guidelines change your behaviour? | Explore opinions of impact of guideline on behaviour |
|  | Do you think the guidelines are achievable? | Do you think of 150 minutes as a threshold? |  |
|  | How does it make you feel hearing this information from a healthcare professional? |  | Explore opinion of delivery of information via healthcare service, as is currently advised by UKCMO |
| Suggested changes to national guideline communication | What type of information relating to physical activity do you think is useful to know? | Is it helpful to know how much? Why?  Is it important to address how to achieve recommendations? Why? | Explore opinions of most valued information |
|  | What is your view on communicating the benefits/risks of physical activity/physical inactivity? |  |  |
|  | Where would you want to hear this information from? | Is there a person or source that it would be most useful to hear this information from?  Is there a beneficial moment to share this information? | Explore opinions of most valued source |

Appendix B – UKCMOPAG Infographic
